# Epidemiology of congenital upper limb anomalies in Korea: A nationwide population-based study

**DOI:** 10.1371/journal.pone.0248105

**Published:** 2021-03-09

**Authors:** Young Ho Shin, Goo Hyun Baek, Ye-Jee Kim, Min-ju Kim, Jae Kwang Kim

**Affiliations:** 1 Department of Orthopaedic Surgery, Asan Medical Center, University of Ulsan College of Medicine, Seoul, Republic of Korea; 2 Department of Orthopaedic Surgery, Seoul National University College of Medicine, Seoul, Republic of Korea; 3 Department of Clinical Epidemiology and Biostatistics, Asan Medical Center, University of Ulsan College of Medicine, Seoul, Republic of Korea; BG Trauma Center Ludwigshafen, GERMANY

## Abstract

This study aimed to analyze the epidemiology of congenital upper limb anomalies (CULA) in Korea. We evaluated the incidence of each type of CULA, the presence of coexisting anomalies and the surgical treatment status in CULA patients. We conducted a retrospective cohort study of patients aged < 1 year between 2007 and 2016 who were registered with CULA in the Health Insurance Review and Assessment Service of Korea. In total, 10,704 patients had CULA, including 6,174 boys (57.7%) and 4,530 girls (42.3%). The mean annual incidence of CULA was 23.5 per 10,000 live births; it was significantly higher in boys than in girls (26.3 vs. 20.5, p < 0.001). Among the four categories of CULA—polydactyly, syndactyly, limb deficiency, and other anomalies—polydactyly was the most common. In total, 4,149 patients (38.8%) had other congenital anomalies and coexisting anomalies of the circulatory system (24.9%) were the most common. In total 4,776 patients (44.6%) underwent operative treatment for CULA within minimum three years of the diagnosis. The proportion of patients who underwent surgical treatment was significantly higher for polydactyly (73.4% vs. 16.8%, p < 0.001) and syndactyly (65.3% vs. 41.5%, p < 0.001), but it was significantly lower in limb deficiency (27.6% vs. 45.4%, p < 0.001) and other anomalies (10.0% vs. 69.8%, p < 0.001) than rest of CULA patients. Among the patients who had operations, 21.5% underwent multiple operations. The proportion of patients who underwent multiple operations was significantly higher in syndactyly (35.6% vs. 18.1%, p < 0.001), but it was significantly lower in polydactyly (4.0% vs. 95.5%, p < 0.001) and other anomalies (17.9% vs. 21.9%, p < 0.001) than rest of CULA patients. These results could provide a basis for estimating the national healthcare costs for CULA and the required number of CULA specialists.

## Introduction

An understanding of the epidemiology of congenital anomalies is important for public health. This information provides a basis for estimating the national healthcare costs and the number of required specialists. In addition, monitoring the changes in the incidence and patterns of congenital anomalies may alert us to new teratogens such as thalidomide in the 1960s [1].

The epidemiology of congenital upper limb anomalies (CULA) has been studied previously, but there were several limitations to consider it as definite epidemiologic information. Some studies have focused on regional, not national populations [2–5], or evaluated the prevalence of CULA, not incidence [3–5], or only examined for one specific anomaly such as limb deficiency [6, 7]. In addition, there are only two old regional studies that have evaluated the epidemiology of CULA in an Asian population [8, 9] and no national studies.

Korea has been implementing a health insurance system for all citizens since 1989. Since the medical data of the whole population in Korea are managed at the Health Insurance Review and Assessment Service (HIRA), the HIRA dataset makes it easy to retrieve and analyze data to understand the medical status of the whole country. In addition, due to the wide coverage of the national insurance system, medical access for Korean citizens is the best among the Organization for Economic Cooperation and Development (OECD) countries [10]. Therefore, we could evaluate the epidemiology of CULA in whole nationwide population by analyzing HIRA data. The purpose of this study was to analyze the epidemiology of CULA in Korea. More specifically, we evaluated the incidence of each type of CULA, the presence of coexisting anomalies and the surgical treatment status in CULA patients.

## Materials and methods

### Data source

In Korea, the National Health Insurance Service (NHIS) covers 100% of the population; 97% have health insurance and 3% have medical aid [11]. All healthcare providers submit claims data for inpatient and outpatient management to the HIRA for reimbursement of medical costs. These include diagnostic codes (classified according to the International Classification of Diseases, 10th revision [ICD-10]), procedure codes, and demographic information. HIRA provides some of this national data to support public policy developments and research activities when requested. This study protocol was exempted for review by the Institutional Review Board of Asan Medical Center (No. 2020–0124) in accordance with the exemption criteria.

### Data collection

We conducted a retrospective cohort study of CULA patients between 2007 and 2016. First, patients aged < 1 year with CULA were identified using ICD-10 codes (Table 1). The ICD-10 codes for CULA were divided into four categories: polydactyly, syndactyly, limb deficiency and other anomalies [12]. For patients who identified several times with the same code, the timing of the first diagnosis was the criterion used for the calculation of annual incidence. If one patient had multiple CULA codes, each code was counted separately for initial analysis, but when calculating the annual incidence of all CULA and each category of anomaly, it was considered as a single case. For example, if one patient was registered with three different codes (e.g., accessory finger(s) (Q690.), accessory thumb(s) (Q691.), and other congenital malformations of upper limb(s) including shoulder girdle (Q740.)), he or she was counted separately for each code incidence, but as a single case for the annual incidence of CULA. For some diagnostic codes, upper and lower extremities were not discriminated. For example, polydactyly unspecified (Q699.), polysyndactly (Q704.), and congenital absence of unspecified limb(s) (Q730.) were considered as CULA codes when they were registered with the procedure codes for radiographs of the upper extremity from clavicle to finger (clavicle: G3101–3105; scapula: G3201–3205; shoulder: G33013305; acromioclavicular joint: G3901–3905; forearm: G6101–6105; elbow: G6201–6205; humerus: G6301–6305; wrist: G6401–6405; hand: G6501–6505; carpal bone: G6601–6605; finger: G8101–8105). The annual incidence of CULA was defined as the proportion of the population who were newly diagnosed with CULA at age < 1 year among the live births during that year. Annual live birth data, including numbers and sex, were acquired from the Korean Statistical Information Service [13]. In addition, other demographic information including sex and insurance type (whether a patient had health insurance or medical aid, which indirectly reflect the social economic status) of each patient were acquired.

**Table 1:** Total number of patients who were registered with each diagnostic code for congenital upper limb anomalies (CULA) in South Korea from 2007 to 2016.

Diagnostic code	Number of patients	Incidence per 10,000 live births (95% CI)
Total	10,704	23.52 (23.08–23.97)
Polydactyly	5,264	11.57 (11.26–11.89)
Q690. Accessory finger(s)	1,545	3.40 (3.23–3.57)
Q691. Accessory thumb(s)	2,424	5.33 (5.12–5.54)
Q699. Polydactyly unspecified*	2,495	5.48 (5.27–5.70)
Syndactyly	1,405	3.09 (2.93–3.25)
Q700. Fused fingers	352	0.77 (0.69–0.86)
Q701. Webbed fingers	236	0.52 (0.45–0.59)
Q704. Polysyndactyly*	435	0.96 (0.87–1.05)
Q709. Syndactyly, unspecified*	613	1.35 (1.24–1.46)
Limb deficiency	490	1.08 (0.98–1.18)
Q710. Congenital complete absence of upper limb(s)	6	0.01 (0.00–0.03)
Q711. Congenital absence of upper arm and forearm with hand present	10	0.02 (0.01–0.04)
Q712. Congenital absence of both forearm and hand	8	0.02 (0.01–0.03)
Q713. Congenital absence of hand and finger(s)	251	0.55 (0.49–0.62)
Q714. Longitudinal reduction defect of radius	40	0.09 (0.06–0.12)
Q715. Longitudinal reduction defect of ulna	5	0.01 (0.00–0.03)
Q716. Lobster–claw hand	23	0.05 (0.03–0.08)
Q718. Other reduction defects of upper limb(s)	118	0.26 (0.21–0.31)
Q719. Reduction defect of upper limb, unspecified	45	0.10 (0.07–0.13)
Q730. Congenital absence of unspecified limb(s)*	16	0.04 (0.02–0.06)
Q731. Phocomelia, unspecified limb(s)*	7	0.02 (0.01–0.03)
Q738. Other reduction of unspecified limb(s)*	9	0.02 (0.01–0.04)
Other anomalies	4,507	9.91 (9.62–10.20)
Q681. Congenital deformity of hand	1,741	3.83 (3.65–4.01)
Q688. Other specified congenital musculoskeletal deformities of U/E	1,592	3.50 (3.33–3.68)
Q740. Other congenital malformations of upper limb(s), including shoulder girdle	980	2.15 (2.02–2.29)
Q743. Arthrogryposis multiplex congenita*	121	0.27 (0.22–0.32)
Q748. Other specified congenital malformations of limb(s)*	114	0.25 (0.21–0.30)
Q749. Unspecified congenital malformation of limb(s)*	160	0.35 (0.30–0.41)

Second, other accompanying congenital anomalies were analyzed for CULA patients. Patients who were diagnosed and registered as having other anomalies within one year of birth, were considered to have other congenital anomalies, classified by the major classification level of ICD-10 codes (Table 2). For congenital anomalies of the musculoskeletal system, patients with anomalies other than CULA were included. The number of patients with other anomalies were evaluated for each type of CULA. If one patient had multiple other anomalies, each anomaly was counted separately according to the major classification level of ICD-10 codes for initial analysis, but when calculating the incidence of other accompanying anomalies in patients with CULA, and each category of CULA, it was considered as a single case. For example, if one patient with thumb polydactyly was registered with two different anomalies including the circulatory system (Q20–28) and the digestive system (Q38–45) within one year after birth, he or she was counted separately for the incidence of each anomaly, but counted as a single case for the incidence of accompanying other anomalies in patients with all CULA and polydactyly.

**Table 2:** Other accompanying congenital anomalies in total and for each category of congenital upper limb anomalies (CULA) in South Korea from 2007 to 2016: Number (incidence (%)).

	Nervus system (Q00–07)	Eye, ear, face and neck (Q10–18)	Circulatory system (Q20–28)	Respiratory system (Q30–34)	Cleft lip and cleft palate (Q35–37)	Digestive system (Q38–45)	Genital organs (Q50–56)	Urinary system (Q60–64)	Musculoskeletal system (Q65–79)*	Other malformations (Q80–89)	Chromosomal abnormalities (Q90–99)	Total
**All patients with CULA**	835 (7.8)	1,213 (11.3)	2,670 (24.9)	607 (5.7)	663 (6.2)	2,103 (19.6)	962 (9.0)	1,396 (13.0)	2,130 (19.9)	889 (8.3)	548 (5.1)	4,149 (38.8)
**Polydactyly**	401 (7.6)	639 (12.1)	1,366 (25.9)	312 (5.9)	336 (6.4)	1,120 (21.3)	510 (9.7)	740 (14.1)	841 (16.0)	414 (7.9)	286 (5.4)	1,843 (35.0)
**Syndactyly**	133 (9.5)	192 (13.7)	392 (27.9)	77 (5.5)	101 (7.2)	324 (23.1)	139 (9.9)	237 (16.9)	337 (24.0)	145 (10.3)	93 (6.6)	614 (43.7)
**Limb deficiency**	57 (11.6)	79 (16.1)	197 (40.2)	43 (8.8)	48 (9.8)	144 (29.4)	68 (13.9)	84 (17.1)	170 (34.7)	65 (13.3)	32 (6.5)	302 (61.6)
**Other anomalies**	370 (8.2)	477 (10.6)	1,060 (23.5)	260 (5.8)	281 (6.2)	813 (18.0)	376 (8.3)	539 (12.0)	1,083 (24.0)	387 (8.6)	218 (4.8)	1,884 (41.8)

*For congenital anomalies of the musculoskeletal system, patients with anomalies other than CULA were assessed.

Third, the surgical treatment status for CULA patients was analyzed. Since the data were collected until the end of 2019, surgical treatments conducted within minimum three years of the initial diagnosis of CULA were included. Patients registered with operation codes under the CULA codes were defined as having surgery for CULA. In the HIRA operation codes, only three codes were disease specified codes for CULA including operation of polydactyly required reconstruction of tendon and bone (N0251), operation of polydactyly required other procedures (N0252), and operation of syndactyly (N0260). The remaining codes were for general bone and soft tissue procedures. Therefore, we identified all operation codes which were possible for surgical treatment of CULA (Table 3). The incidence of surgical treatment, the time to initial operation from diagnosis, and the number of operations were analyzed for each type of CULA.

**Table 3:** Available operation codes of Health Insurance Review and Assessment Service of South Korean for congenital upper limb anomalies (CULA).

	Description	Codes
**Disease specified codes**	Operation for polydactyly	N0251 (construction of tendon or bone), N0252 (others)
	Operation for syndactyly	N0260
**General bone and soft tissue procedures**	Release of scar contracture	N0241
	Release of scar contracture and skin graft	Full thickness: N0242 (<25 cm^2^), N0243 (≥25 cm^2^)
		Split thickness (face or joint): N0244 (<25 cm^2^), N0245 (≥ 25 cm2)
		Split thickness (others): N0246 (<25 cm^2^), N0247 (25–99 cm^2^), NA241 (100–399 cm^2^), NA242 (400–899 cm^2^), NA243 (≥900 cm^2^)
	Release of scar contracture and flap operation	N0249
	Osteotomy	N0302 (upper or lower extremity), N0316 (carpal or tarsal), N0317 (metacarpal, metatarsal, finger, or toe)
	Osteotomy and internal fixation	N0304 (radius or ulna), N0306 (humerus), N0307 (radius and ulna), N0318 (carpal or tarsal), N0319 (metacarpal, metatarsal, finger, or toe)
	Ostectomy	N0311
	Bone graft	N0312
	Disarticulation of extremities	N0563 (shoulder), N0565 (elbow, wrist, or ankle), N0566 (finger or toe)
	Amputation of extremities	N0573 (upper arm, forearm, or lower leg), N0574 (hand or foot), N0575 (finger or toe)
	Excision of carpal or tarsal bone	N0610
	Resection arthroplasty	N0722 (shoulder, knee, elbow, wrist, or ankle), N0723 (finger or toe)
	Arthrodesis	N0733 (elbow, wrist, or ankle), N0734 (finger or toe), N0738 (shoulder),
	Open reduction of dislocation	N0752 (shoulder), N0753 (elbow), N0755 (wrist or ankle), N0756 (finger or toe)
	Closed reduction of dislocation	N0762 (shoulder), N0763 (elbow or knee), N0764 (wrist, ankle, finger, or toe), N0765 (radial head subluxation)
	Mechanical correction for deformity	N0792 (deformity of extremity)
	Manipulative correction for deformity	N0804
	Reconstruction of tendon and ligament	N0931 (simple: resection, suture, or release), N0932 (complex: graft, transfer, or reconstruction with allograft)
	Tenolysis	N0941
	Vascularized osteocutaneous free flap	N1583 (vascularized bone graft), N1584 (vascularized osteocutaneous graft), N1585 (pedicled vascularized bone graft)
	Autogenous fat graft or dermofat graft	NX021

### Statistical analysis

Continuous data were presented as mean ± standard deviation (SD) or median and interquartile range (IQR), and categorical data are presented as numbers and percentages. We calculated the annual incidence of CULA (per 10,000 live births) in boys and girls assuming a Poisson distribution. Poisson regression analysis was used to analyze the trends in annual incidence of overall CULA, CULA in each sex, and in the four categories of CULA. The chi-square test was used to compare the proportion of patients in each category who had other accompanying congenital anomalies and those who underwent operative treatment and multiple operations with the rest of CULA patients. P < 0.05 was interpreted as statistically significant. All statistical analyses were performed using the SAS Enterprise Guide software version 7.1 (SAS Institute, Inc., Cary, NC, USA).

## Results

### Annual incidence of congenital upper limb anomalies

**Fig 1:**
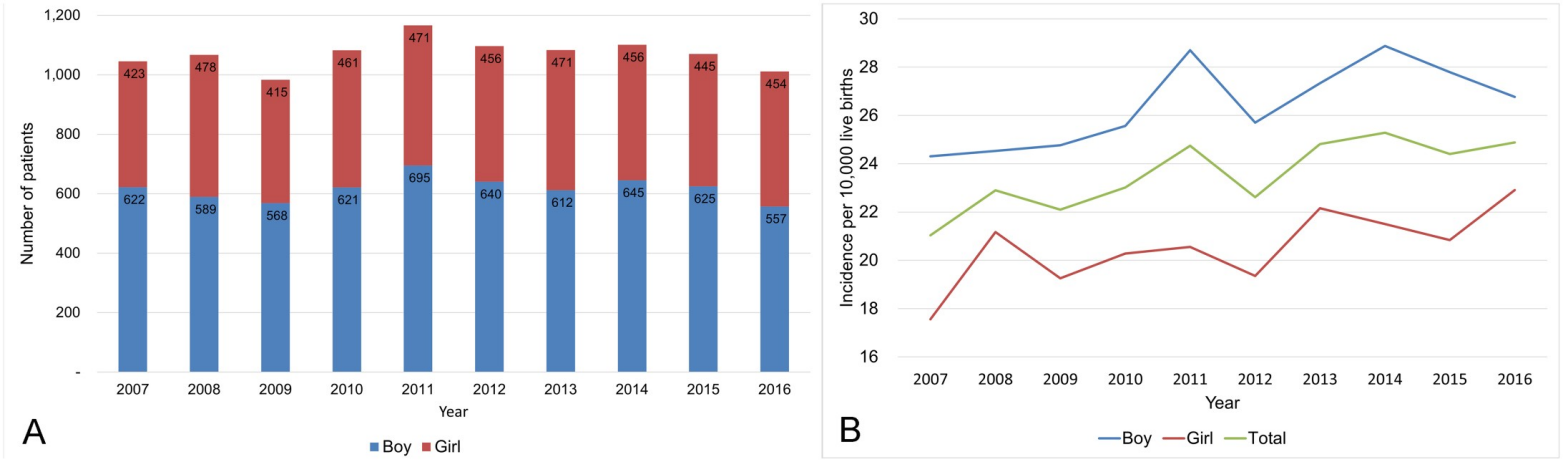
Annual number (A) and incidence (B) of congenital upper limb anomalies (CULA) in South Korea from 2007 to 2016.

A total of 10,704 patients were registered with CULA from 2007 to 2016, including 6,174 boys (57.7%) and 4,530 girls (42.3%), and a total of 4,550,102 live births were registered in the same period (Table 4). The mean annual incidence of CULA was 23.5 per 10,000 live births, and it was significantly higher in boys (26.3 per 10,000 live births) than girls (20.5 per 10,000 live births) (p < 0.001) (Table 5). Among the total 10,704 patients, 10,561 patients (98.7%) had health insurance and 143 patients (1.3%) had medical aid. The Poisson regression analysis showed that the annual incidence of CULA increased during the study period (incidence rate ratio (IRR), 1.017; 95% CI, 1.009–1.025; p < 0.001). This increase was observed in both boys (IRR, 1.016; 95% CI, 1.006–1.026; p = 0.021) and girls (IRR, 1.018; 95% CI, 1.006–1.031; p = 0.036) (Fig 1).

**Table 4:** Annual number of total and each category of congenital upper limb anomalies (CULA) in South Korea from 2007 to 2016.

	2007	2008	2009	2010	2011	2012	2013	2014	2015	2016	Total
**All patients with CULA**	1,045	1,067	983	1,082	1,166	1,096	1,083	1,101	1,070	1,011	10,704
**Polydactyly**	585	552	485	517	573	529	556	489	527	451	5,264
**Syndactyly**	148	135	130	132	137	152	159	140	142	130	1,405
**Limb deficiency**	54	54	39	44	56	62	47	38	52	44	490
**Other anomalies**	375	426	413	487	495	466	416	521	447	461	4,507

**Table 5:** Annual incidence of congenital upper limb anomalies (CULA) in South Korea from 2007 to 2016.

	Total			Boy			Girl		
Year	No. of patients	No. of population	Incidence per 10,000 live births (95% CI)	No. of patients	No. of population	Incidence per 10,000 live births (95% CI)	No. of patients	No. of population	Incidence per 10,000 live births (95% CI)
**2007**	1,045	496,822	21.0 (19.78–22.35)	622	255,872	24.31 (22.44–26.30)	423	240,950	17.56 (15.92–19.31)
**2008**	1,067	465,892	22.9 (21.55–24.32)	589	240,119	24.53 (22.59–26.59)	478	225,773	21.17 (19.32–23.16)
**2009**	983	444,849	22.1 (20.74–23.52)	568	229,351	24.77 (22.77–26.89)	415	215,498	19.26 (17.45–21.20)
**2010**	1,082	470,171	23.0 (21.66–24.43)	621	242,901	25.57 (23.59–27.66)	461	227,270	20.28 (18.47–22.22)
**2011**	1,166	471,265	24.7 (23.34–26.20)	695	242,121	28.70 (26.61–30.92)	471	229,144	20.55 (18.74–22.50)
**2012**	1,096	484,550	22.6 (21.30–24.00)	640	248,958	25.71 (23.75–27.78)	456	235,592	19.36 (17.62–21.22)
**2013**	1,083	436,455	24.8 (23.36–26.34)	612	223,883	27.34 (25.21–29.59)	471	212,572	22.16 (20.20–24.25)
**2014**	1,101	435,435	25.3 (23.81–26.82)	645	223,356	28.88 (26.69–31.19)	456	212,079	21.50 (19.57–23.57)
**2015**	1,070	438,420	24.4 (22.97–25.91)	625	224,906	27.79 (25.65–30.06)	445	213,514	20.84 (18.95–22.87)
**2016**	1,011	406,243	24.9 (23.38–26.47)	557	208,064	26.77 (24.59–29.09)	454	198,179	22.91 (20.85–25.12)

No. (number), CI (confidence intervals).

**Fig 2:**
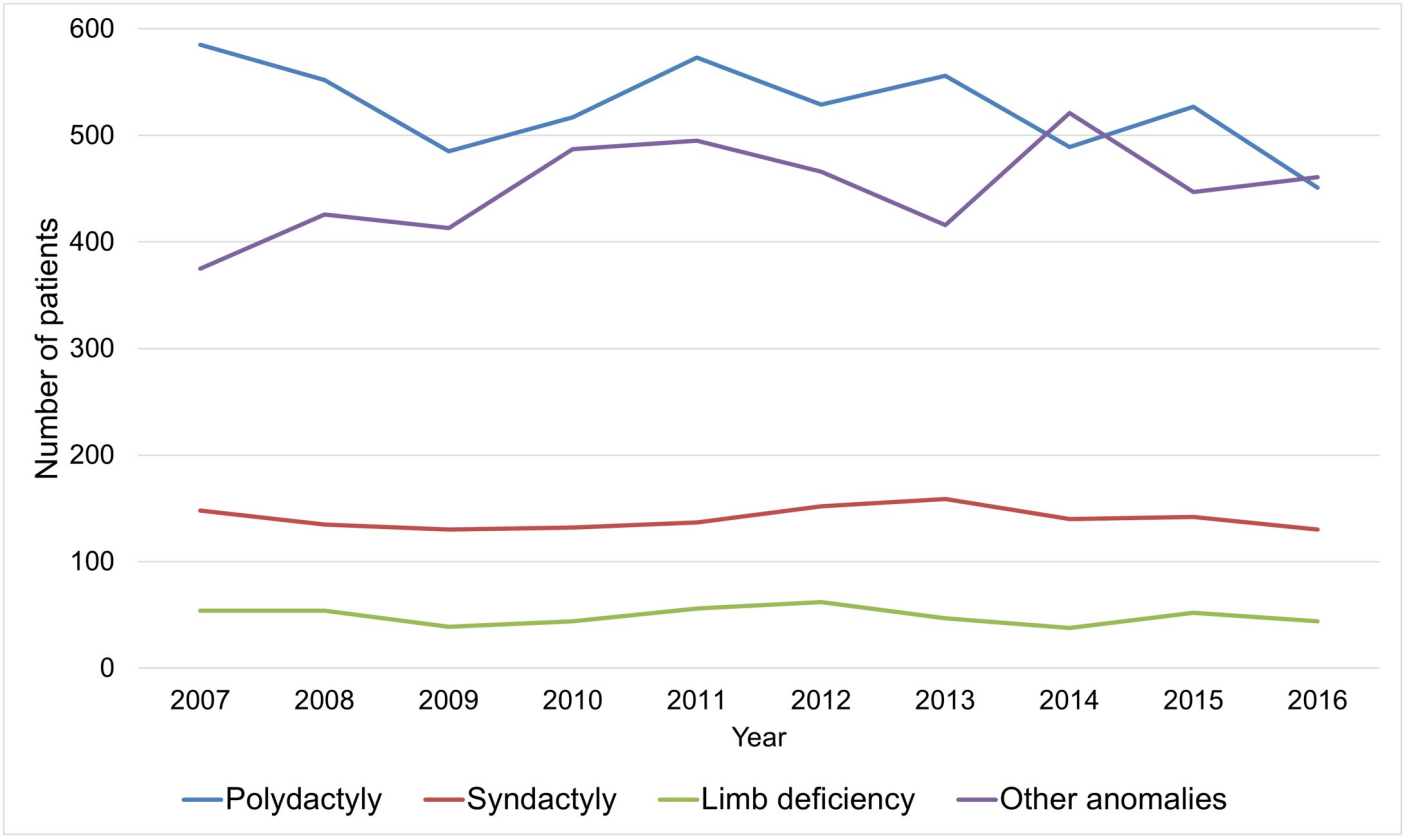
Annual number of patients with congenital upper limb anomalies (CULA) in South Korea from 2007 to 2016 according to the categories.

Among the four ICD-10 codes categories, polydactyly (5,264 patients, 49.2%) was most common and followed by other anomalies (4,507 patients, 42.1%), syndactyly (1,405 patients, 13.1%), and limb deficiency (490 patients, 4.6%). The Poisson regression analyses showed that the annual incidence of polydactyly (IRR, 1.000; 95% CI, 0.988–1.013; p = 0.951) and limb deficiency (IRR, 1.004; 95% CI, 0.974–1.034; p = 0.810) were not significantly changed during the study period. However, the annual incidence of syndactyly (IRR, 1.017; 95% CI 1.003–1.031; p = 0.018) and other anomalies (IRR, 1.033; 95% CI, 1.013–1.052; p = 0.001) were significantly increased during the study period (Fig 2).

#### Other accompanying congenital anomalies in patients with congenital upper limb abnormality

**Fig 3:**
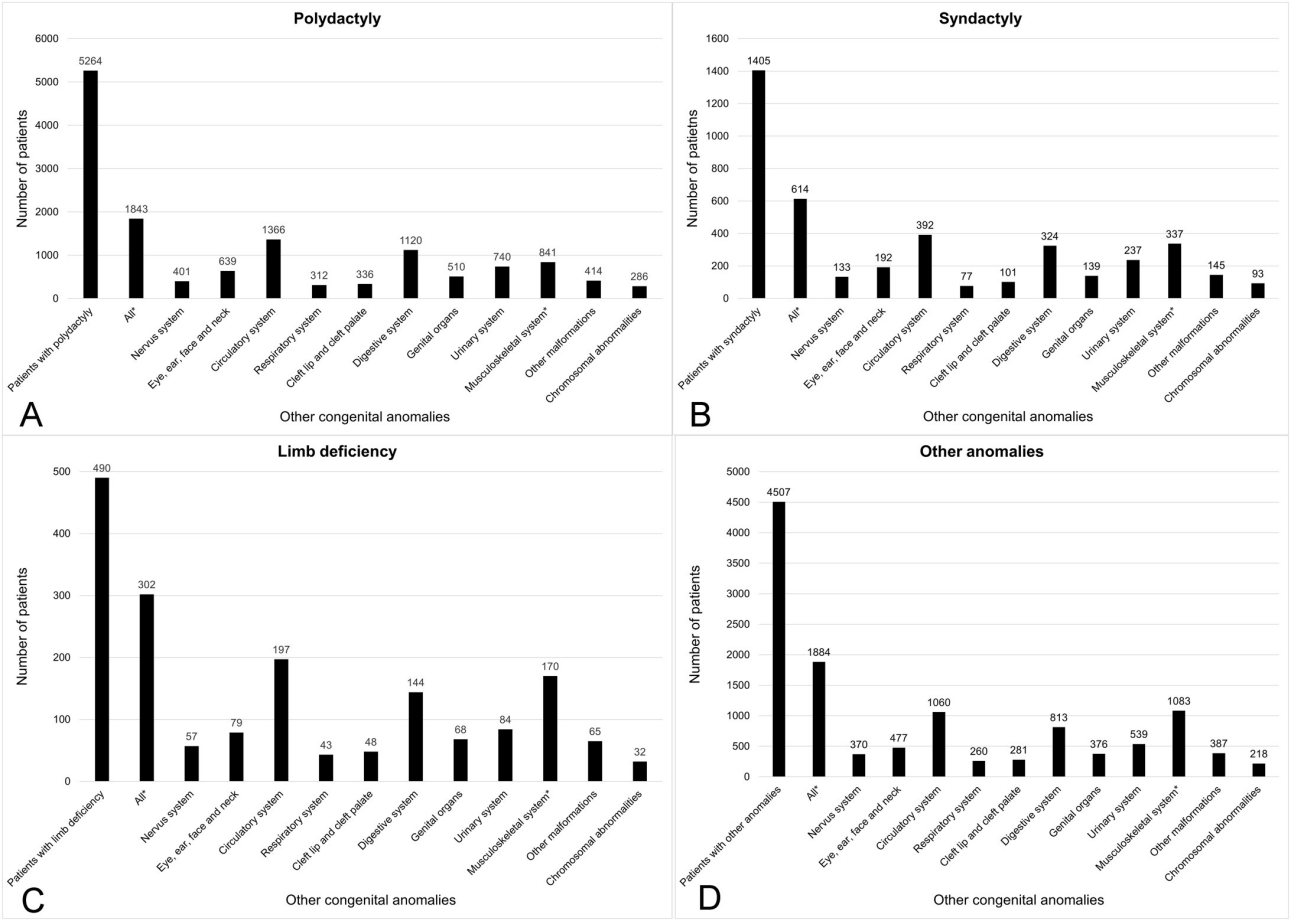
Other accompanying congenital anomalies which accompanied with congenital upper limb anomalies (CULA) in South Korea from 2007 to 2016 according to the categories: (A) polydactyly, (B) syndactyly, (C) limb deficiency, and (D) other anomalies.

Among the 10,704 patients with newly diagnosed CULA, 4149 patients (38.8%) had other congenital anomalies. Congenital anomalies in the circulatory system were the most common (2670 patients, 24.9%), followed by congenital anomalies in the musculoskeletal system other than CULA (2130 patients, 19.9%) and congenital anomalies in the digestive system (2103 patients, 19.6%) (Table 2). The proportion of patients with other accompanying congenital anomalies was described in Fig 3 according to category. Among the four categories, other congenital anomalies was significantly higher in limb deficiency (61.6% vs. 37.7%, p < 0.001), syndactyly (43.7% vs. 38.0%, p < 0.001), and other anomalies (41.8% vs. 36.5%, p < 0.001), but significantly lower in polydactyly (35.0% vs. 42.4%, p < 0.001) than rest of CULA patients.

#### Surgical treatment status for congenital upper limb abnormality

Among the 10,704 patients with newly diagnosed CULA, 4,776 patients (44.6%) underwent operative treatment for CULA within minimum three years of the diagnosis. The proportion of patients who had surgical treatments was described in Table 6. Among the four categories, surgical treatment rate was significantly higher in polydactyly (73.4% vs. 16.8%, p < 0.001), and syndactyly (65.3% vs. 41.5%, p < 0.001), but significantly lower in limb deficiency (27.6% vs. 45.4%, p < 0.001), and other anomalies (10.0% vs. 69.8%, p < 0.001) than rest of CULA patients.

**Table 6:** The proportion of patients who underwent operative treatment for congenital hand and upper extremity anomaly (CULA) in South Korea from 2007 to 2016 within minimum three years of diagnosis.

Diagnostic code	Proportion (%)
Total	44.6
Polydactyly	73.4
Q690. Accessory finger(s)	77.5
Q691. Accessory thumb(s)	79.6
Q699. Polydactyly unspecified*	72.4
Syndactyly	65.3
Q700. Fused fingers	70.2
Q701. Webbed fingers	61.0
Q704. Polysyndactyly*	80.7
Q709. Syndactyly, unspecified*	63.0
Limb deficiency	27.6
Q710. Congenital complete absence of upper limb(s)	16.7
Q711. Congenital absence of upper arm and forearm with hand present	20.0
Q712. Congenital absence of both forearm and hand	12.5
Q713. Congenital absence of hand and finger(s)	26.7
Q714. Longitudinal reduction defect of radius	32.5
Q715. Longitudinal reduction defect of ulna	20.0
Q716. Lobster–claw hand	47.8
Q718. Other reduction defects of upper limb(s)	32.2
Q719. Reduction defect of upper limb, unspecified	44.4
Q730. Congenital absence of unspecified limb(s)*	0.0
Q731. Phocomelia, unspecified limb(s)*	0.0
Q738. Other reduction of unspecified limb(s)*	22.2
Other anomalies	10.0
Q681. Congenital deformity of hand	15.6
Q688. Other specified congenital musculoskeletal deformities of U/E	4.0
Q740. Other congenital malformations of upper limb(s), including shoulder girdle	9.0
Q743. Arthrogryposis multiplex congenita*	14.0
Q748. Other specified congenital malformations of limb(s)*	12.3
Q749. Unspecified congenital malformation of limb(s)*	18.8

**Fig 4:**
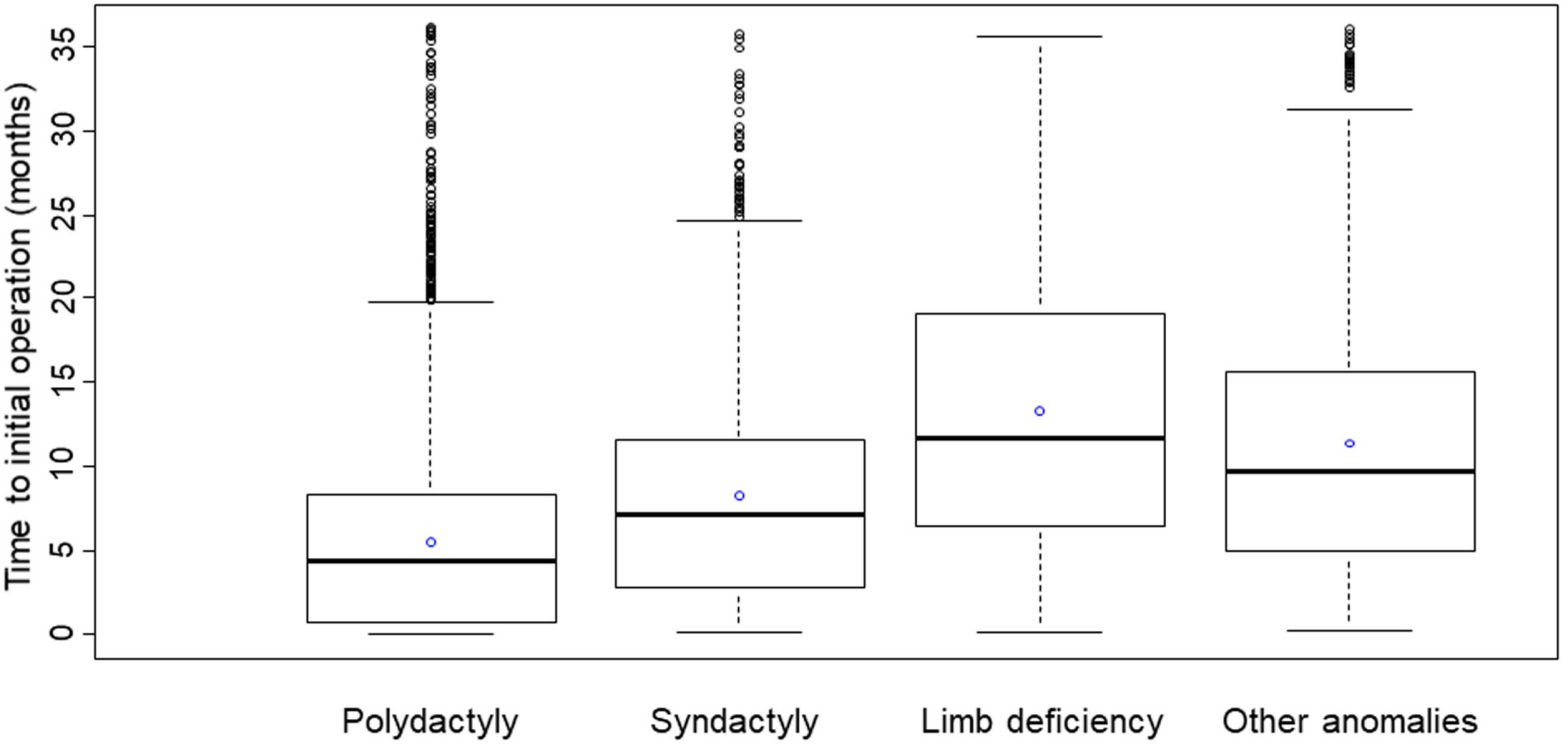
The median time from diagnosis to initial operation from the diagnosis for patients who had surgical treatment for congenital upper limb anomalies (CULA) in South Korea from 2007 to 2016 according to the categories; empty blue circle: Mean time.

**Fig 5:**
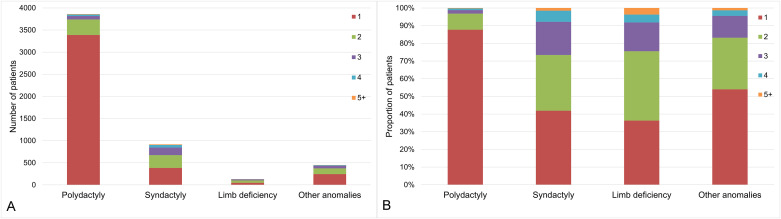
The number of operations for patients who had surgical treatment for congenital upper limb anomalies (CULA) in South Korea from 2007 to 2016 according to the categories: (A) number of patients and (B) proportion of patients according to the operation numbers.

For the 4,776 patients who had surgical treatments, the median time to the initial operation from diagnosis was 5.1 months (IQR, 0.9–9.4 months). Among the four categories, it was longest in limb deficiency, median value 11.7 months (IQR, 6.4–19.1 months) and shortest in polydactyly, median value 4.4 months (IQR, 0.7–8.4 months) (Fig 4). Among the 4,776 patients who underwent operations, 3750 patients (78.5%) underwent a single operation, but 1026 patients (21.5%) underwent multiple operations for CULA. Among the four categories, the portion of patients who had multiple operations was significantly higher in syndactyly (35.6% vs. 18.1%, p < 0.001), but significantly lower in polydactyly (4.0% vs. 95.5%, p < 0.001) and other anomalies (17.9% vs. 21.9%, p < 0.001) than rest of CULA patients (Fig 5).

## Discussion

The overall incidence of CULA in our study (23.5 per 10,000 live births) was similar to that reported in previous studies. In a total population study of Western Australia spanning 11 years, Giele et al. reported the prevalence of CULA as 19.8 per 10,000 live births [2]. In a total population study of Stockholm, Sweden over 11 years, Ekblom et al. reported the incidence of CULA as 21.5 in 10,000 live births [4]. In a study of the New York Congenital Malformations Registry spanning 18 years, Goldfarb et al. reported the prevalence as 27.2 per 10,000 live births [3]. These differences could originate from the differences in data sources, racial compositions of the populations studied, and whether incidence or prevalence was measured. In two previous studies [2, 4], more boys than girls had CULA, however a statistical significant difference was revealed for the first time in our study. The annual live birth rates for Korea slightly decreased, but the annual numbers of CULA was constant or slightly increased during the study period. This resulted in the statistically significant increase in the annual incidence of CULA regardless of sex. The increased mean maternal age (30.6 years in 2007 and 32.4 years in 2016) and decreased mortality rate of newborns (34 per 10,000 live births in 2007 and 28 per 10,000 live births in 2016) [13] which mean higher survival rate of babies with multiple anomalies could attribute to the rise in incidence of CULA [2]. In addition, this increasing trend may reflect improved detection and reporting of CULA, or an increase of exogenous teratogens in our environment [6].

Polydactyly was the most common category of CULA, but the data did not allow for identification of the anomaly location. Although the ‘other anomalies’ category was the second most common category which included unspecified various anomalies, syndactyly was the second most common as a single anomaly entity. This finding is the generally accepted ranking of CULA incidence [3, 14]. The incidence of upper limb deficiency was lower than the incidence in Northern Europe cohorts [4, 6, 7], with half of the patients having an absence of hand and finger(s). We think that these differences in CULA compositions originates from the ethnic differences of each cohort.

Among the patients with newly diagnosed CULA, 38.8% had other congenital anomalies in our cohort. This portion is lower than the 46% in the Western Australian cohort [2], but higher than the 23.1% in the Swedish cohort [4]. Regarding the type of accompanying congenital anomalies, syndromic anomalies were the commonest or second commonest cause in previous studies [2, 4]; however, it was less common in our cohort. We think that these discrepancies originate from data collection methods. In previous studies, all available medical records and radiographs were reviewed by specialists and in cases where the CULA was a part of syndromic anomalies it was classified as ‘accompanying a syndromic anomaly’ and not ‘other specific organ anomalies’. However, in our cohort, only the registered data were analyzed, and we could not review the detailed medical records or diagnosis for CULA patients. As all healthcare providers could submit the patients`data in our cohort, there is the possibility that specific organ anomalies were registered simultaneously with a syndromic anomaly. Among the four categories, a higher portion of limb deficiency patients had other congenital anomalies than other CULA categories. Therefore, when we treat patients with limb deficiency, we should consider the possibility of other anomalies and their general medical condition.

As most surgical treatments for CULA are initiated within two or three years after birth [15, 16], we think that the observation period of this study (minimum three years after the diagnosis) would include most of the surgically treated CULA patients. Although the portion of surgically treated patients were higher in polydactyly and syndactyly than the rest of CULA patients, it was not over 90% in both categories. This may relate to the underreporting of rudimentary type polydactyly, which could be removed with ligation or simple excision at an outpatient clinic or neonatal nursery instead of official surgery under anesthesia [17]. In addition, some cases of partial syndactyly which do not show functional disability could be observed without operation, while in some complex syndactyly cases surgery could be contraindicated if there was a risk of further functional impairment [3, 18]. For limb deficiency and other anomalies, the portion of surgically treated patients were lower than for the rest of the CULA patients. This phenomena could be explained by the limited role of surgical treatment in limb deficiency patients [6, 7] and ‘other anomalies’ include anomalies which do not impair the function and thus do not require surgical treatment, such as clinodactyly, brachydactyly, minor type clasped or hypoplastic thumb [16]. The portion of patients who had multiple operations was significantly higher in syndactyly than for the rest of the CULA patients. This may relate to the higher reoperation rate of syndactyly due to web creep and deviation of the divided digit [19], and the multiple operations required for multiple webs or for operations on both hands [20].

Our study has several limitations similar to those found in any registry study. First, this is an imperfect registry for identifying all CULA correctly as confirmation of CULA often requires clinical and radiological assessment by specialists such as congenital hand surgeons [3]. Therefore, some patients could be registered with different codes at different times. We believe that the polydactyly, syndactyly, and limb deficiency data are reliable as these cases were easy to identify. In addition, we are confident total CULA incidence data is accurate as we removed repetitive data for the same patient. In contrast, patients with ‘other anomalies’ are the least reliable data due to various and less straightforward diagnoses. Second, most studies stratified their cases with known CULA classification systems such as the International Federation of Societies for Surgery of the Hand (IFSSH) classification [2, 21] or the Oberg, Manske, and Tonkin (OMT) classification [4, 5]. We could not apply these classification systems to our cohort because the information required was not captured in our database. This limits the direct comparison of results between epidemiological studies. However, because this study covered the whole national population and most of the CULA diagnoses would be registered by non-specialists, registration and analysis of ICD-10 data is suitable for this type of study, as these are familiar to all healthcare providers. Third, the time limitations of this study could under-estimate the incidence of CULA and their surgical treatments. Some CULA such as clinodactyly, brachymesophalangy, and the Sprengel deformity could be detected after one year of age. Limiting the time for surgery to be performed to three years from diagnosis may be too short to include all multiple operations.

## Conclusions

The incidence of CULA to be 23.5 per 10,000 for 10 years and the incidence increased slightly over a 10-year period. Among the four categories: polydactyly, syndactyly, limb deficiency, and other anomalies, polydactyly was the most common type of CULA. A total of 38.8% of patients with CULA had other congenital anomalies with anomalies of the circulatory system being the most associated. A total of 44.6% of patients with CULA underwent operative treatment for CULA and the proportion was significantly higher in polydactyly and syndactyly than in the rest of the CULA patients. Among the patients who underwent operations, 21.5% of the patients underwent multiple operations. The portion of patients who had multiple operations was significantly higher in syndactyly than the rest of the CULA patients. These results could facilitate an understanding of the epidemiology of CULA in an Asian population and provide a basis for estimating the national healthcare costs for CULA and the number of specialists needed to treat CULA.

## Supporting information

S1 Table Annual number of registered each diagnostic codes for congenital upper limb anomalies (CULA) in South Korea from 2007 to 2016. (DOCX)

S2 Table Other congenital anomalies which accompanied with each diagnostic codes for congenital upper limb anomalies (CULA) in South Korea from 2007 to 2016. (DOCX)
