# Supplementary material for: Epidemiology of congenital upper limb anomalies in Korea: A nationwide population-based study
Source: PLoS One. 2021 Mar 9;16(3):e0248105. doi: 10.1371/journal.pone.0248105 (PMC7943020; doi:10.1371/journal.pone.0248105)
Supplement: S1 Table — (DOCX) [file pone.0248105.s001.docx]

S1 Table. Annual number of registered each diagnostic codes for congenital upper limb anomalies (CULA) in South Korea from 2007 to 2016

| Diagnostic code | 2007 | 2008 | 2009 | 2010 | 2011 | 2012 | 2013 | 2014 | 2015 | 2016 | Total |
| --- | --- | --- | --- | --- | --- | --- | --- | --- | --- | --- | --- |
| Annual number of CULA | 1,045 | 1,067 | 983 | 1,082 | 1,166 | 1,096 | 1,083 | 1,101 | 1,070 | 1,011 | 10,704 |
| Polydactyly | 585 | 552 | 485 | 517 | 573 | 529 | 556 | 489 | 527 | 451 | 5,264 |
| Q690. Accessory finger(s) | 127 | 138 | 112 | 166 | 193 | 176 | 176 | 166 | 157 | 134 | 1,545 |
| Q691. Accessory thumb(s) | 256 | 240 | 221 | 213 | 251 | 244 | 254 | 252 | 261 | 232 | 2,424 |
| Q699. Polydactyly unspecified* | 326 | 314 | 275 | 275 | 281 | 228 | 244 | 180 | 198 | 174 | 2,495 |
| Syndactyly | 148 | 135 | 130 | 132 | 137 | 152 | 159 | 140 | 142 | 130 | 1,405 |
| Q700. Fused fingers | 28 | 36 | 26 | 32 | 37 | 37 | 33 | 37 | 49 | 37 | 352 |
| Q701. Webbed fingers | 9 | 22 | 20 | 16 | 28 | 24 | 19 | 34 | 31 | 33 | 236 |
| Q704. Polysyndactyly* | 41 | 33 | 40 | 54 | 35 | 50 | 54 | 43 | 45 | 40 | 435 |
| Q709. Syndactyly, unspecified* | 78 | 76 | 54 | 51 | 64 | 64 | 72 | 48 | 52 | 54 | 613 |
| Limb deficiency | 54 | 54 | 39 | 44 | 56 | 62 | 47 | 38 | 52 | 44 | 490 |
| Q710. Congenital complete absence of upper limb(s) | 0 | 2 | 1 | 0 | 0 | 3 | 0 | 0 | 0 | 0 | 6 |
| Q711. Congenital absence of upper arm and forearm with hand present | 1 | 2 | 2 | 2 | 0 | 1 | 0 | 1 | 1 | 0 | 10 |
| Q712. Congenital absence of both forearm and hand | 1 | 0 | 1 | 0 | 0 | 2 | 2 | 1 | 1 | 0 | 8 |
| Q713. Congenital absence of hand and finger(s) | 28 | 31 | 23 | 22 | 31 | 33 | 22 | 15 | 28 | 18 | 251 |
| Q714. Longitudinal reduction defect of radius | 5 | 4 | 2 | 3 | 4 | 5 | 4 | 3 | 4 | 6 | 40 |
| Q715. Longitudinal reduction defect of ulna | 0 | 0 | 0 | 0 | 2 | 0 | 1 | 1 | 0 | 1 | 5 |
| Q716. Lobster–claw hand | 3 | 2 | 1 | 2 | 4 | 1 | 1 | 5 | 3 | 1 | 23 |
| Q718. Other reduction defects of upper limb(s) | 8 | 7 | 4 | 9 | 12 | 13 | 17 | 13 | 21 | 14 | 118 |
| Q719. Reduction defect of upper limb, unspecified | 5 | 7 | 4 | 8 | 3 | 5 | 1 | 1 | 4 | 7 | 45 |
| Q730. Congenital absence of unspecified limb(s)* | 4 | 0 | 1 | 1 | 6 | 1 | 1 | 0 | 1 | 1 | 16 |
| Q731. Phocomelia, unspecified limb(s)* | 2 | 1 | 1 | 0 | 1 | 0 | 1 | 1 | 0 | 0 | 7 |
| Q738. Other reduction of unspecified limb(s)* | 2 | 1 | 0 | 0 | 0 | 4 | 1 | 0 | 1 | 0 | 9 |
| Other anomalies | 375 | 426 | 413 | 487 | 495 | 466 | 416 | 521 | 447 | 461 | 4,507 |
| Q681. Congenital deformity of hand | 95 | 116 | 179 | 186 | 176 | 200 | 169 | 199 | 215 | 206 | 1,741 |
| Q688. Other specified congenital musculoskeletal deformities of U/E | 154 | 174 | 146 | 208 | 193 | 134 | 110 | 196 | 139 | 138 | 1,592 |
| Q740. Other congenital malformations of upper limb(s), including shoulder girdle | 76 | 86 | 66 | 83 | 117 | 120 | 126 | 113 | 76 | 117 | 980 |
| Q743. Arthrogryposis multiplex congenita* | 11 | 20 | 11 | 8 | 20 | 11 | 7 | 15 | 11 | 7 | 121 |
| Q748. Other specified congenital malformations of limb(s)* | 21 | 20 | 12 | 12 | 12 | 14 | 9 | 3 | 6 | 5 | 114 |
| Q749. Unspecified congenital malformation of limb(s)* | 33 | 31 | 13 | 16 | 16 | 10 | 11 | 8 | 12 | 10 | 160 |

*For some diagnosis codes which upper and lower extremities are not discriminated, we considered those codes as CULA when they were registered with the procedure codes for radiographs of upper extremity from clavicle to finger.
