# Supplementary material for: Epidemiology of congenital upper limb anomalies in Korea: A nationwide population-based study
Source: PLoS One. 2021 Mar 9;16(3):e0248105. doi: 10.1371/journal.pone.0248105 (PMC7943020; doi:10.1371/journal.pone.0248105)
Supplement: S2 Table — (DOCX) [file pone.0248105.s002.docx]

S2 Table. Other congenital anomalies which accompanied with each diagnostic codes for congenital upper limb anomalies (CULA) in South Korea from 2007 to 2016

|  | Nervus system (Q00–07) | Eye, ear, face and neck (Q10–18) | Circulatory system (Q20–28) | Respiratory system (Q30–34) | Cleft lip and cleft palate (Q35–37) | Digestive system (Q38–45) | Genital organs (Q50–56) | Urinary system (Q60–64) | Musculoskeletal system (Q65–79)** | Other malformations (Q80–89) | Chromosomal abnormalities (Q90–99) | Total |
| --- | --- | --- | --- | --- | --- | --- | --- | --- | --- | --- | --- | --- |
| All patients with CULA | 835 | 1213 | 2670 | 607 | 663 | 2103 | 962 | 1396 | 2130 | 889 | 548 | 4149 |
| Polydactyly | 401 | 639 | 1366 | 312 | 336 | 1120 | 510 | 740 | 841 | 414 | 286 | 1843 |
| Q690. Accessory finger(s) | 97 | 153 | 344 | 74 | 81 | 267 | 114 | 175 | 204 | 105 | 60 | 509 |
| Q691. Accessory thumb(s) | 137 | 252 | 534 | 111 | 105 | 410 | 183 | 253 | 297 | 148 | 98 | 752 |
| Q699. Polydactyly unspecified* | 217 | 330 | 700 | 171 | 193 | 589 | 285 | 403 | 454 | 227 | 173 | 917 |
| Syndactyly | 133 | 192 | 392 | 77 | 101 | 324 | 139 | 237 | 337 | 145 | 93 | 614 |
| Q700. Fused fingers | 26 | 37 | 86 | 15 | 21 | 73 | 34 | 49 | 81 | 27 | 16 | 150 |
| Q701. Webbed fingers | 25 | 37 | 69 | 11 | 23 | 60 | 34 | 46 | 73 | 32 | 17 | 111 |
| Q704. Polysyndactyly* | 26 | 36 | 84 | 13 | 17 | 65 | 27 | 43 | 67 | 28 | 16 | 147 |
| Q709. Syndactyly, unspecified* | 64 | 92 | 177 | 42 | 47 | 144 | 59 | 113 | 164 | 72 | 50 | 277 |
| Limb deficiency | 57 | 79 | 197 | 43 | 48 | 144 | 68 | 84 | 170 | 65 | 32 | 302 |
| Q710. Congenital complete absence of upper limb(s) | 1 | 1 | 2 | 0 | 1 | 3 | 1 | 1 | 2 | 1 | 0 | 4 |
| Q711. Congenital absence of upper arm and forearm with hand present | 1 | 2 | 5 | 2 | 1 | 3 | 1 | 3 | 3 | 3 | 0 | 7 |
| Q712. Congenital absence of both forearm and hand | 1 | 1 | 1 | 0 | 1 | 1 | 1 | 3 | 2 | 0 | 1 | 4 |
| Q713. Congenital absence of hand and finger(s) | 24 | 37 | 88 | 19 | 20 | 70 | 39 | 44 | 93 | 31 | 19 | 141 |
| Q714. Longitudinal reduction defect of radius | 8 | 7 | 20 | 3 | 4 | 13 | 9 | 11 | 17 | 6 | 4 | 31 |
| Q715. Longitudinal reduction defect of ulna | 0 | 0 | 0 | 0 | 1 | 1 | 0 | 0 | 3 | 1 | 0 | 4 |
| Q716. Lobster–claw hand | 1 | 5 | 5 | 1 | 5 | 2 | 1 | 2 | 7 | 3 | 0 | 12 |
| Q718. Other reduction defects of upper limb(s) | 17 | 22 | 68 | 13 | 11 | 38 | 18 | 24 | 30 | 17 | 6 | 82 |
| Q719. Reduction defect of upper limb, unspecified | 4 | 6 | 13 | 2 | 6 | 9 | 0 | 4 | 17 | 5 | 0 | 27 |
| Q730. Congenital absence of unspecified limb(s)* | 3 | 3 | 8 | 0 | 3 | 6 | 2 | 2 | 6 | 2 | 2 | 11 |
| Q731. Phocomelia, unspecified limb(s)* | 0 | 2 | 5 | 2 | 1 | 3 | 2 | 2 | 3 | 4 | 1 | 6 |
| Q738. Other reduction of unspecified limb(s)* | 1 | 1 | 3 | 2 | 2 | 5 | 0 | 1 | 2 | 0 | 1 | 7 |
| Other anomalies | 370 | 477 | 1060 | 260 | 281 | 813 | 376 | 539 | 1083 | 387 | 218 | 1884 |
| Q681. Congenital deformity of hand | 161 | 231 | 483 | 119 | 124 | 373 | 173 | 255 | 437 | 168 | 103 | 779 |
| Q688. Other specified congenital musculoskeletal deformities of U/E | 132 | 158 | 374 | 92 | 97 | 293 | 127 | 197 | 447 | 147 | 75 | 722 |
| Q740. Other congenital malformations of upper limb(s), including shoulder girdle | 60 | 73 | 151 | 34 | 42 | 110 | 62 | 69 | 143 | 46 | 32 | 296 |
| Q743. Arthrogryposis multiplex congenita* | 19 | 18 | 43 | 15 | 18 | 29 | 16 | 20 | 65 | 22 | 14 | 93 |
| Q748. Other specified congenital malformations of limb(s)* | 9 | 8 | 21 | 6 | 6 | 16 | 7 | 9 | 28 | 7 | 6 | 40 |
| Q749. Unspecified congenital malformation of limb(s)* | 17 | 21 | 48 | 12 | 13 | 40 | 20 | 24 | 47 | 21 | 7 | 78 |

*For some diagnosis codes which upper and lower extremities are not discriminated, we considered those codes as CULA when they were registered with the procedure codes for radiographs of upper extremity from clavicle to finger.

**For congenital anomalies of musculoskeletal system, patients with anomalies other than CULA were assessed
